# A Fragment of Apolipoprotein E4 Leads to the Downregulation of a CXorf56 Homologue, a Novel ER-Associated Protein, and Activation of BV2 Microglial Cells

**DOI:** 10.1155/2019/5123565

**Published:** 2019-05-06

**Authors:** Tanner B. Pollock, Jacob M. Mack, Ryan J. Day, Noail F. Isho, Raquel J. Brown, Alexandra E. Oxford, Brad E. Morrison, Eric J. Hayden, Troy T. Rohn

**Affiliations:** ^1^Department of Biological Sciences, Boise State University, Science/Nursing Building, Room 228, Boise, Idaho 83725, USA; ^2^University of Washington, School of Medicine, Seattle, WA 98195, USA

## Abstract

Despite the fact that harboring the apolipoprotein E4 (*APOE4*) allele represents the single greatest risk factor for late-onset Alzheimer's disease (AD), the exact mechanism by which apoE4 contributes to disease progression remains unknown. Recently, we demonstrated that a 151 amino-terminal fragment of apoE4 (nApoE4_1-151_) localizes within the nucleus of microglia in the human AD brain, suggesting a potential role in gene expression. In the present study, we investigated this possibility utilizing BV2 microglia cells treated exogenously with nApoE4_1-151_. The results indicated that nApoE4_1-151_ leads to morphological activation of microglia cells through, at least in part, the downregulation of a novel ER-associated protein, CXorf56. Moreover, treatment of BV2 cells with nApoE4_1-151_ resulted in a 68-fold increase in the expression of the inflammatory cytokine, TNF*α*, a key trigger of microglia activation. In this regard, we also observed a specific binding interaction of nApoE4_1-151_ with the TNF*α* promoter region. Collectively, these data identify a novel gene-regulatory pathway involving CXorf56 that may link apoE4 to microglia activation and inflammation associated with AD.

## 1. Introduction

Alzheimer's disease (AD) currently has a significant global impact. In the USA alone, over 5.7 million Americans suffer from this progressive, irreversible brain disorder that destroys memory and thinking skills [1]. A central tenet underlying AD is chronic inflammation that may contribute to the underlying pathology and neurodegeneration. Evidence documented for the past 30 years has proven the existence of inflammation in AD, including activated microglia within and surrounding senile plaques [2–5]. Despite this wealth of evidence, the stimuli that led to microglia activation and subsequent inflammation in AD are not well described. One possible connection linking these two events may be the apoE4 protein. The *APOE4* allele represents the single greatest risk factor for late-onset AD, and inheritance of one copy of the *APOE4* allele increases Alzheimer's disease (AD) risk fourfold, while two copies raises the risk tenfold [6]. A recent study linking apoE4 to neuroinflammation in AD demonstrated that apoE4-expressing microglia exhibit higher innate immune reactivity after lipopolysaccharide treatment and this in turn may promote neuroinflammation in AD [7].

We recently documented the presence of an amino-terminal fragment of apoE4 1-151 (nApoE4_1-151_) within the nucleus of microglia cells in the human AD brain [8]. *In vitro*, exogenous treatment of BV2 microglial cells with nApoE4_1-151_ led to uptake and trafficking to the nucleus [8]. In the present study, we sought to examine the functional consequences of nuclear localization of nApoE4_1-151_ with a working hypothesis that this fragment acts as a transcription factor leading to gene expression changes within microglia. Our results indicated that nApoE4_1-151_ does regulate the expression of several genes including a novel, previously uncharacterized gene, *CXorf56*. These findings have potential broad implications implicating apoE4 to dementia risk perhaps through microglia activation and enhanced inflammation in the AD brain.

## 2. Materials and Methods

### 2.1. Materials

The anti-CXorf56 rabbit polyclonal antibody was purchased from Thermo Fisher Scientific (Rockford, IL). Besides human CXorf56, this antibody has the highest antigen sequence identity to mouse (100%) and rat (100%) CXorf56. The mouse monoclonal anti-calnexin antibody was purchased from Abcam Inc. (Cambridge, MA). MitoTracker Deep Red FM was purchased from Thermo Fisher Scientific (Rockford, IL). Construction and purification of the amino-terminal fragment 1-151 for apoE4 (nApoE4_1-151_) were contracted out to GenScript (Piscataway, NJ). The anti-beta-actin monoclonal antibody was purchased from ABclonal (Woburn, MA).

### 2.2. Cell Culture of BV2 Cells

BV2, murine microglial cells, were maintained at 37°C and 6% CO_2_ in a humidified incubator. Cells were maintained in RPMI 1640 media (HyClone) supplemented with 10% standard fetal bovine serum (HyClone), 10% Cellgro MEM Nonessential Amino Acid (Corning), and 10% penicillin-streptomycin (HyClone). Cells were cultured in 50 ml T25 flasks. For experimentation, cells were grown on BioCoat poly-D-lysine glass multiwell culture slides (Corning). All supplies were purchased from Thermo Fisher Scientific Inc. (Waltham, MA). Treatment of BV2 cells was undertaken by incubation with nApoE4_1-151_ 25 *μ*g/ml for either 5 hours to assess mRNA expression or 24 hours prior to immunocytochemical or Western blot studies.

### 2.3. Chromatin Immunoprecipitation (ChIP-seq)

Chromatin immunoprecipitation (ChIP-seq) was employed to determine what DNA sequences the nApoE4_1-151_ fragment may be binding to directly following treatment of BV2 cells. ChIP-seq was performed according to the manufacturer's instructions (Abcam's high-sensitivity ChIP kit (product number: ab185913). nApoE4_1-151_ pulled-down DNA sequences were then outsourced (DNA Integrated Technologies) to be sequenced. Numerous controls were included including a positive control antibody (RNA polymerase II), a negative control nonimmune IgG, and GAPDH primers to demonstrate the efficacy of the kit reagents and protocol.

### 2.4. CXorf56 and TNF*α* Quantitative PCR

Primers were designed to specifically amplify a portion of either the CXorf56 or TNF*α* genes. Serine/arginine-rich splicing factor 11 (SFRS11) and EH domain-binding protein 1 (EHBP), two ultraconserved elements that have invariant copy number in mice, were used as reference genes. All primers were synthesized by Integrated DNA Technologies (Coralville, IA). For TNF*α*, the forward sequence was ACGGCATGGATCTCAAAGAC and the reverse was AGATAGCAAATCGGCTGACG [9]. Primer efficiencies (*E*%) were confirmed to be between 90 and 110%. Primers were confirmed to be specific based upon melting profiles (Table 1).

The total volume for each reaction was 20 *μ*l and included 10 *μ*l Fast EvaGreen qPCR Master Mix (Biotium Inc., Ca, USA), 1 *μ*l of each appropriate primer (10 *μ*M), 4 *μ*l of water, and 4 *μ*l of template cDNA. Each PCR reaction also included a reverse transcription negative control to confirm the absence of genomic DNA in triplicate and a nontemplate negative control to confirm the absence of primer dimerization in triplicate. Real-time qPCR was run on a LightCycler 96 (Roche, Basel, Switzerland). The cycling conditions were 1 cycle of denaturation at 95°C for 3 min, followed by 40 cycles of amplification (95°C for 30 sec, 55°C for 30 sec, and 68°C for 30 sec) and one cycle of product melting (95°C for 10 sec, 65°C for 60 sec, and 97°C for 1 sec). All samples were amplified in triplicate, and the Cq value for each reaction was determined by the LightCycler 96 SW1.1. Relative differences in expression between treatments were determined by the LightCycler 96 SW1.1 and confirmed with the ΔΔCt method.

### 2.5. Double-Stranded Small Interfering RNA Synthesis and Transfection

Double-stranded small interfering RNAs (siRNAs) were synthesized by Integrated DNA Technologies (Coralville, IA). Two different siRNAs were synthesized to target CXorf56 mRNA and termed siRNA(2) and siRNA(3), respectively. A third control siRNA was designed as a negative control and consisted of a scrambled sequence. BV2 microglia cells were approximately 60% confluent prior to transfection. Transfection was achieved by combining 25 *μ*l of Opti-MEM Reduced Serum Media (Thermo Fisher Scientific), 0.75 *μ*l of Lipofectamine RNAiMAX Transfection Reagent (Thermo Fisher Scientific), and 1.25 pmol of the appropriate siRNA and pipetting the mixture directly onto the plated cells in 48-well plates. A TYE 563-labeled siRNA was used as a positive transfection control, a HPRT-s1 siRNA was used as an endogenous gene positive control, and a scrambled siRNA was used as a negative control. Treatments were for 5 hours to assess CXorf56 mRNA downregulation or 24 hours to assess protein levels.

### 2.6. Total RNA Extraction and cDNA Synthesis

Total RNA was extracted from cells with the Direct-zol RNA MicroPrep Kit (Zymo Research Corp., CA, USA) according to manufacturer's instructions. Genomic DNA was eliminated using TURBO DNAse as described by the manufacturer (Life Technologies, CA). RNA quality was assessed using spectrophotometry and gel electrophoresis. Total cDNA was generated from 1 *μ*g of total RNA using qScript cDNA SuperMix (QuantaBio, MA, USA). Prior to use in qPCR, cDNA was diluted 1 : 2 with water.

### 2.7. Confocal Microscopy

Following treatment studies, BV2 cells were fixed by incubating cells in 4% paraformaldehyde for 23 minutes. For antibody labeling, cells were washed with 0.1 M Tris-buffered saline (TBS), pH 7.4, and pretreated with 3% hydrogen peroxide in 10% methanol to block endogenous peroxidase activity. Slides were subsequently washed in TBS with 0.1% Triton X-100 (TBS-A) and then blocked for thirty minutes in TBS-A with 3% bovine serum albumin (TBS-B). Slides were further incubated overnight at room temperature with the anti-His rabbit antibody (1 : 2,000). Following two washes with TBS-A and a wash in TBS-B, slides were incubated with the anti-rabbit HRP-conjugated secondary antibody. Visualization was accomplished by using a tyramide signal amplification kit (Molecular Probes, Eugene, OR) consisting of Alexa Fluor 488-labeled tyramide (green, Ex/Em = 495/519). Slides were mounted using ProLong Gold Antifade Mountant with DAPI (Molecular Probes).

### 2.8. Microsome Isolation and Western Blot Analysis

ER-enriched fractions from BV2 microglia cells were prepared using a microsome isolation kit (BioVision, Milpitas, CA) according to the manufacturer's instructions. Western blot analysis was performed as previously described [10]. Proteins were separated by 15% SDS-PAGE and transferred to nitrocellulose. Transferred slabs were stained in Coomassie blue to verify equal loading between samples. Membranes were incubated in a CXorf56 antibody (1 : 500) or a calnexin antibody (1 : 100) overnight at 4°C, and primary antibodies were visualized using the goat anti-rabbit HRP-linked secondary antibody, incubated for 1 hour at room temperature (1 : 5,000) (Jackson's Laboratory, West Grove, PA), followed by ECL detection. To confirm equal protein loading, Western blot analysis was also carried out using a beta-actin antibody at 1 : 50,000). Densitometry analysis was performed using the Image Studio software, version 5.2.5 (LI-COR Biosciences).

### 2.9. Quantification of Morphological Changes and Statistical Analysis

NeuronJ is distributed as a plugin for ImageJ, free image analysis software distributed by the National Institutes of Health. Semiautomated tracing with NeuronJ has been shown to be as accurate as both fully manual [11] and fully automated tracing [12]. BV2 microglia cells were treated as described above with siRNAs for 24 hours. Following treatments, two-dimensional 20x images were obtained using bright-field microscopy. Images from each well were taken in a random location and converted to the proper format for use with the NeuronJ software. Following conversion, the number of cells and number of pseudopods in each image were manually counted. To assess the length of the pseudopods, the neuron tracing feature of NeuronJ was employed. After pseudopods were traced, a text file containing measurements of the lengths of all the pseudopods was exported to Excel for analysis. Statistical differences in this study were determined using Student's two-tailed *t*-test employing Microsoft Office Excel.

### 2.10. DNA Mobility Shift Assays

The binding reactions were created by combining 2 *μ*l of 10x binding buffer (Thermo Scientific Cat. #20148), 1 *μ*l of 50% glycerol, 1 *μ*l of 100 mM MgCl_2_, 1 *μ*l of 1 *μ*g/*μ*l poly(dI · dC), 1 *μ*l of 1% NP-40, and 20 fmol of TNF*α* promoter region DNA. The murine TNF*α* promoter region DNA was synthesized and biotinylated at the 5′ end by Integrated DNA Technologies Inc. utilizing the following sequence: ATGCTTGTGTGTCCCCAACTTTCCAAATCCCCGCCCCCGCGATGGAGAAGAAACCGAGACAGAAGGTGCAGGGCCCACTACCGCTTCCTCCAGATGAGCTCATGGGTTTCTCCACCAAGGAAGTTTTCCGCTGGTTGAATGATTCTTTCCCCGCCCTCCTCTCGCCCCAGGGACATATAAAG GCAGTTGTTGGCACACCA. Finally, either 3.6 *μ*g of nApoE4_1-151_ protein fragment or 3.6 *μ*g of full-length ApoE4 protein was added with the addition of nuclease-free water to each reaction to bring the final volume to 20 *μ*l. All reactions were incubated at room temperature for twenty minutes.

Following preincubation of DNA with protein, a 5% TBE precast polyacrylamide gel (Bio-Rad Cat. #4565013) was prepared with 0.5x TBE as the loading buffer. The gel was preelectrophoresed at 100 V for 30 minutes. 5 *μ*l of 5x loading buffer (Thermo Scientific Cat. #20148) was mixed into each reaction; 20 *μ*l of each mixture was loaded, and reactions were separated at 100 V. Following separation, reactions were transferred at 100 V for 30 minutes onto a Biodyne™ B Precut Modified Nylon Membrane, 0.45 *μ*m (Thermo Scientific Cat. #77016). Following the transfer, the DNA on the membrane was cross-linked at a distance of approximately 0.5 cm from the membrane for 5 minutes with a 254 nm handheld UV lamp. The Biotin-labeled DNA was detected using the Chemiluminescent Nucleic Acid Detection Module (Thermo Scientific Cat. #89880) according to the manufacturer's instructions.

### 2.11. Cytokine ELISA Assays

To quantify the levels of secreted TNF-alpha or IL-1beta in BV2 cells, mouse TNF-alpha or IL-1beta Quantikine ELISA kits from R&D Systems was employed according to the manufacturer's instructions. Briefly, 50 *μ*l of standard, control, or sample was added in duplicate to ELISA plates, incubated at room temperature for 2 hours followed by 5 washes in wash buffer. Next, 100 *μ*l of conjugate was added to each well, sealed, and incubated at room temperature for 2 hours followed by a wash step. 100 *μ*l of substrate solution was added for 30 minutes followed by 100 *μ*l of stop solution. The ELISA plate was read at 450 nm, and concentrations (pg/ml) were determined using a standard curve consisting of either recombinant mouse TNF-alpha or IL-1beta.

## 3. Results

### 3.1. CXorf56 Is an ER-Associated Protein

**Figure 1:**
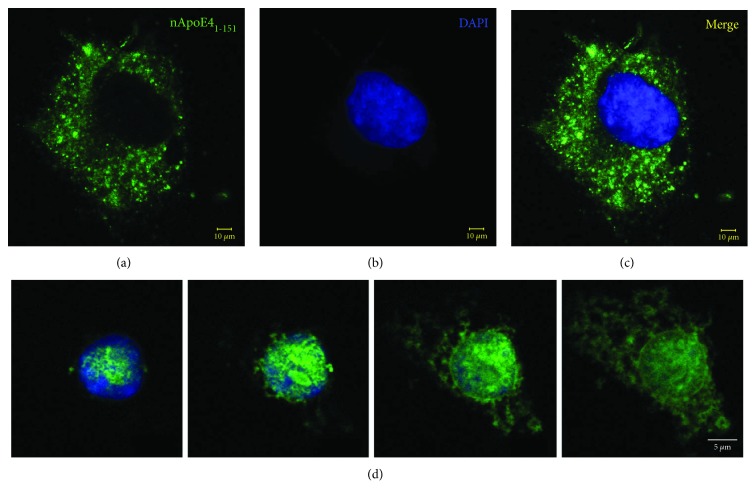
Nuclear localization of an amino-terminal fragment of apoE4 is confirmed in microglia cells but not in astrocytes. (a–c) U87 cells, representing an astrocytic cell line, were plated on glass chamber slides in normal growth media and treated for 24 hours with the nApoE4_1-151_ fragment. Following treatment, cells were fixed and immunocytochemistry was carried out using an anti-His rabbit, polyclonal antibody at 1 : 2,000, followed by the HRP-conjugated secondary antibody at 1 : 200 (see Materials and Methods for details). Under these experimental conditions, although there was evidence of the cytoplasmic uptake of the fragment (a), little nuclear localization of the nApoE4_1-151_ fragment was observed following treatment (c, merge). Scale bars represent 10 *μ*m. (d) A representative set of images showing nuclear localization in the microglial cell line, BV2, following exogenous treatment with the nApoE4_1–151_ fragment. BV2 microglial cells were placed on glass chamber slides in normal growth media and treated for 24 hours with the nApoE4_1–151_ fragment. Following treatment, cells were fixed and immunocytochemistry was carried out as described above. Double-label immunofluorescence confocal z-stacks were acquired to detect nApoE4_1–151_ (green) together with DAPI (blue). The merged images indicated the strong nuclear and cytoplasmic presence of the amino-terminal fragment following extracellular incubation of BV2 cells.

The purpose of the current study was to elucidate the possible functional consequences of nuclear trafficking by nApoE4_1-151_ with our major hypothesis being the fragment acts as an enhancer or a repressor of gene transcription. In the CNS, both microglia and astrocytes synthesize and secrete apoE4 [13, 14]. In a previous study, we documented the presence of a 17 kDa amino-terminal fragment of apoE4 in the nucleus of microglia in human postmortem AD brain sections but found no evidence for this fragment in the nucleus of astrocytes [8]. In addition, exogenous treatment of BV2 microglia cells with the corresponding protein fragment (nApoE4_1-151_) led to uptake and trafficking to the nucleus [8, 15]. To confirm these findings, BV2 cells representing microglia and U87 cells representing astrocytes were treated with or without nApoE4_1-151_ and labeled with an anti-His antibody to track the uptake and localization of the fragment following treatment. As shown in Figure 1, although both cell lines took up the fragment from the surrounding media, nuclear localization was only evident within the BV2 microglia cells (Figure 1(d)). Based on these results, all experiments were performed on BV2 microglia cells due to the fact that nuclear localization of nApoE4_1-151_ would be necessary *a priori* in order for the fragment to regulate gene transcription directly.

As an initial approach, experiments were undertaken using DNA harvested from BV2 microglia cells and chromatin immunoprecipitation (ChIP-seq) analyses to determine what DNA sequences the nApoE4_1-151_ fragment may be binding to directly. Using this approach, a DNA sequence pulled down by immobilized nApoE4_1-151_ was mapped to a 5′ region on chromosome 18 designated Gm8181 in the murine genome (BV2 cells are of murine origin). This DNA sequence was pulled down on three separate occasions employing chromatin immunoprecipitation. Other sequences that were also pulled down were too degraded to confirm sequence identity. Examination of this sequence revealed an uncharacterized gene on human chromosome X termed *CXorf56*. Although the two genes are named differently in the mouse and human, the proteins are virtually identical with there being one amino acid out of 222 that is different between the two species (99.5% sequence identity). Therefore, for clarification purposes, we will refer to the mouse gene as the *CXorf56 homologue* and the mouse protein as CXorf56.

**Figure 2:**
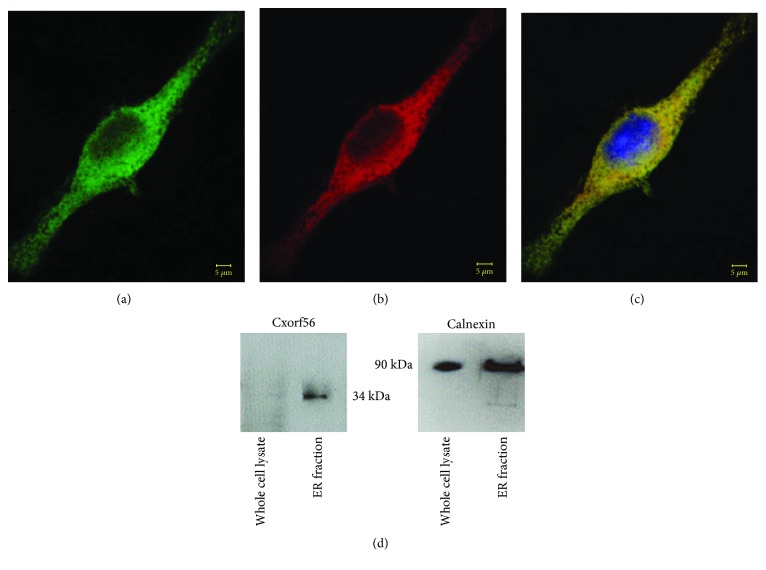
Subcellular localization of the CXorf56 protein to the ER in microglial, BV2 cells. (a–c) Representative images from confocal immunofluorescence in BV2 cells utilizing an anti-CXorf56 antibody (a, green) and an anti-calnexin antibody, an ER-specific marker (b, red), with the overlap image shown in (c) (yellow). DAPI nuclear labeling in blue is shown in (c). A strong perinuclear colocalization between these two antibodies was evident (c). (d) Western blot analysis was carried out using BV2 whole cell lysates (left lanes) or enriched ER fractions (right lanes), and transferred proteins were probed with either the CXorf56 antibody (left panel, 1 : 500) or with the ER marker, calnexin (right panel, 1 : 1,000). The results indicated a 34 kDa immunoreactive band by the CXorf56 antibody in the ER fraction to which calnexin was also immunolabeled (right panel, 90 kDa band). Data are representative of three independent experiments.

Although the *CXorf56* gene has recently been linked to a form of intellectual disability [16], the subcellular localization and function of the expressed protein in the CNS are currently not known. Thus, we performed confocal immunofluorescence studies in microglia cells using an antibody specific to the mouse CXorf56 protein to determine its possible cellular localization. Perinuclear cytoplasmic labeling of this antibody was evident in BV2 microglia cells. Double-label confocal microscopy studies ruled out a staining in mitochondria as evidenced by the lack of colocalization of the anti-CXorf56 antibody with known mitochondrial markers (Fig.  S1). In contrast, a strong colocalization of CXorf56 with calnexin, a specific endoplasmic reticulum (ER), was evident following confocal microscopy analysis (Figures 2(a)–2(c)). To confirm these immunocytochemistry findings, Western blot analysis was performed in whole-cell BV2 extracts or ER-enriched fractions. ER-enriched fractions were validated following immunoblotting with calnexin (Figure 2(d), right panel). Immunoblotting with the anti-CXorf56 antibody revealed a specific band in the ER-enriched fraction corresponding to the correct predicted molecular weight of CXorf56 at 34 kDa (Figure 2(d), left panel). Additional experiments utilizing mouse liver microsomes that are enriched with ER confirmed the high expression of CXorf56 within the ER (Fig.  S2). Taken together, these results identify CXorf56 as an ER-specific protein.

### 3.2. The CXorf56 Protein Is Required to Maintain Microglia Cells in a Quiescent Morphological Phenotype

**Figure 3:**
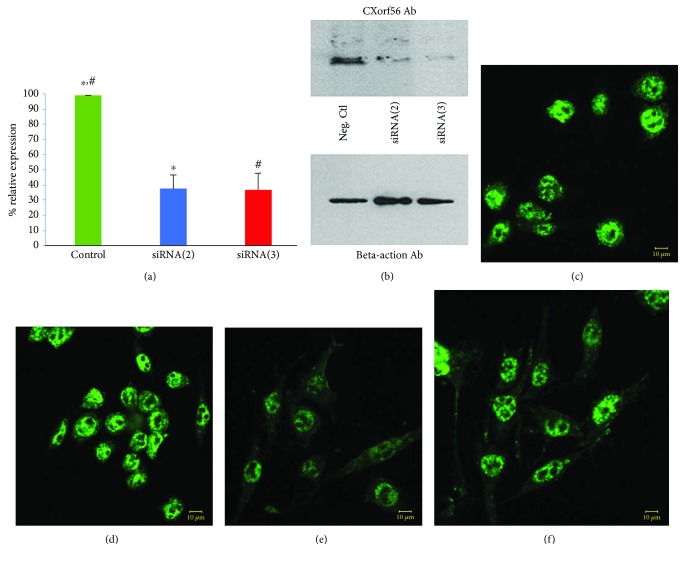
Confirmation of CXorf56 knockdown in BV2 microglial cells following treatment with siRNAs. (a) BV2 cells were treated for 5 hours with a scrambled siRNA sequence (Neg. siRNA Ctl, green bar) or two designed sequences (siRNA2, blue bar or siRNA3, red bar) targeted to the CXorf56 open reading frame. Following treatment, total RNA was extracted and RT qPCR was performed. In contrast to the negative siRNA control, siRNA(2) and siRNA(3) led to a 40% and 58% downregulation, respectively, of CXorf56 mRNA. ∗ denotes significant difference between scrambled negative control and siRNA(2), *p* = 0.0003. # denotes significant difference between negative control and siRNA(3), *p* = 0.0005. (b) Western blot analysis utilizing an anti-CXorf56 antibody (1 : 500) confirmed the decreased expression of the CXorf56 protein following treatment of BV2 cells for 24 hrs with siRNA2 (middle lane) or siRNA3 (far right lane). Beta-actin was employed as a loading control utilizing an anti-beta-actin antibody (1 : 50K), bottom panel. Data are representative of three independent experiments ± S.D. (c–f) Representative confocal immunofluorescence images utilizing an anti-CXorf56 antibody in BV2 cells following treatment with siRNAs for 24 hours with nontreated cells (c), negative siRNA control (d), siRNA(2) (e), and siRNA(3) (f). A decrease in staining intensity with the anti-CXorf56 antibody was observed following treatment with siRNA2 (e) and to a lesser extent with siRNA3 (f). Note also the apparent change in morphology following treatment with extended, bipolar pseudopods in (e) and (f).

To assess a possible function for CXorf56, we employed double-stranded small interfering RNAs (siRNAs) to induce short-term silencing of the CXorf56 gene expression. For these experiments, we designed three different siRNAs: a scrambled siRNA sequence that would serve as a negative control and two siRNAs (siRNA(2) and siRNA(3)) designed to specifically target CXorf56 mRNA for degradation. BV2 microglia cells were treated for 5 hours with either one of these constructs followed by real-time quantitative PCR (qPCR) using SFSR11 and EHBP as internal controls for expression (see Materials and Methods for details). Control experiments indicated that >90% of BV2 cells were successfully transfected following treatment of cells with siRNA constructs. In contrast to the negative siRNA control, treatment with siRNA(2) or (3) led to a 40% and 50% downregulation, respectively, of CXorf56 mRNA (Figure 3(a)). It is noteworthy that increasing concentrations of siRNA above what is shown in Figure 3 did not lead to a greater knockdown of CXorf56 mRNA. At this time, it is not known why we were not able to achieve a greater degree of knockdown but it could be related to a number of factors including proper cellular localization, incorrect 3D structure, or inaccessibility due to bound proteins. Verification of CXorf56 protein knockdown was obtained following Western blot analysis (Figure 3(b)). Confocal microscopy using anti-CXorf56 demonstrated a decrease in the staining intensity as compared to nontreated controls or BV2 cells treated with the negative siRNA control construct (Figures 3(c)–3(f)). Interesting, in addition to a decrease in CXorf56 staining intensity, we also observed a morphological change following CXorf56 knockdown. As depicted in Figures 3(e) and 3(f), microglia cells became elongated and bipolar in appearance.

**Figure 4:**
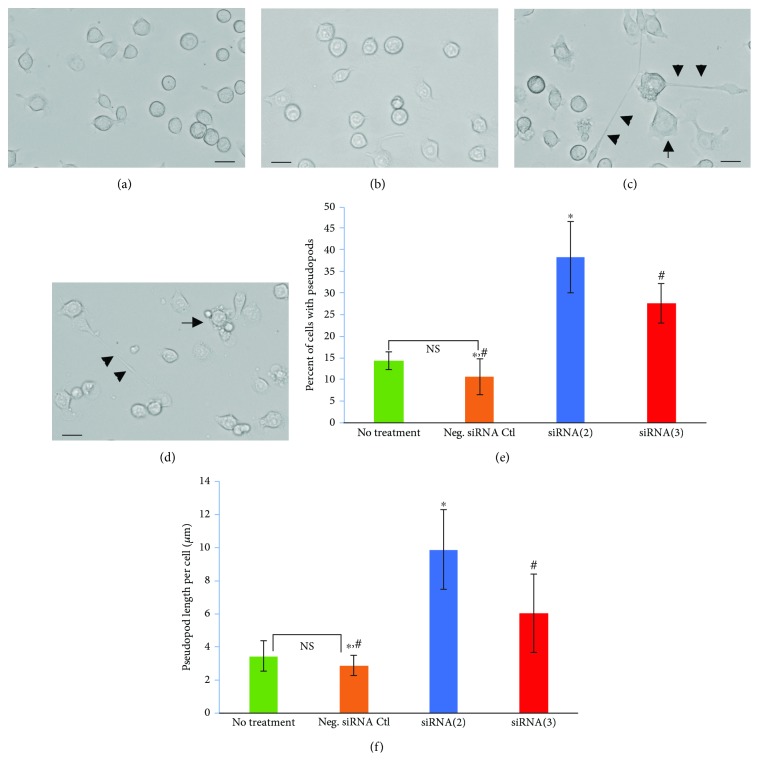
Downregulation of the CXorf56 protein leads to morphological changes in BV2 microglia cells. (a–d) BV2 cells were untreated (a) or treated for 5 hours with scrambled negative control siRNA (b), siRNA2 (c), or siRNA3 targeting the open reading frame CXorf56 (d). Following treatment, bright-field, phase-contrast images were captured. In contrast to untreated or cells treated with the siRNA negative control, cells treated with targeted siRNAs led to changes in morphology from round, compact cells to cells that were flattened and extended long pseudopods (arrowheads, c and d) and or were highly vacuolized (arrows, c and d). Scale bars represent 10 *μ*m. (e, f) Quantification using the ImageJ software showing percent of cells with pseudopods was significantly increased following treatment with either siRNA2 or 3 (e, blue and red bars) as was the length of pseudopods per cell (f, blue and red bars). In (e), ∗ denotes significant difference between scrambled siRNA negative control (orange bar) and siRNA(2) (blue bar), *p* = 0.002; # denotes significant difference between scrambled siRNA negative control (orange bar) and siRNA(3) (red bar), *p* = 0.009. In (f), ∗ denotes significant difference between scrambled siRNA negative control (orange bar) and siRNA(2) (blue bar), *p* = 0.002; # denotes significant difference between scrambled siRNA negative control (orange bar) and siRNA(3) (red bar), *p* = 0.009. Data in (e) and (f) are representative of 4 independent experiments ± S.E.M.

To further characterize a potential morphological role for CXorf56, we carried out bright-field, phase-contrast microscopy with quantitative analysis using the ImageJ software. Visually, knockdown by either siRNA(2) or (3) resulted in cells extending out long pseudopods (arrowheads, Figure 4(d)). Cells also appeared flatter with bright, vesicular structures within the cytoplasm (arrow, Figure 4(d)). A quantitative analysis indicated a significant increase in both the percent of microglia with pseudopods (Figure 4(e)) as well as the overall length of pseudopods per cell (Figure 4(f)). Taken together, these data suggest that the knockdown of the CXorf56 protein leads to a morphological shift to an activated state that is characterized by extension of pseudopods as well as in increase in vacuolar appearance [17–19].

### 3.3. An Amino-Terminal Fragment of apoE4 Leads to Microglia Activation

**Figure 5:**
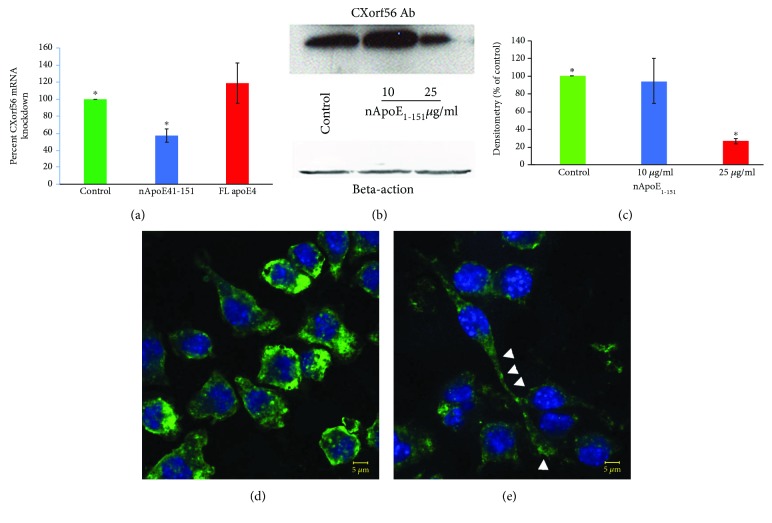
Treatment of BV2 microglial cells with an amino-terminal fragment of apoE4 leads to the downregulation of CXorf56. (a) BV2 cells were treated for 5 hours with 25 *μ*g/mg nApoE4_1-151_ (blue bar) or with an equivalent concentration of full-length apoE4 (red bar). Following treatment, RNA was extracted and RT qPCR was performed. Data indicated a significant decrease in the expression of the CXorf56 homologue following treatment, *p* = 0.0002, while full-length apoE4 had no significant effect. (b, c) Western blot analysis utilizing an anti-CXorf56 antibody (1 : 500) confirmed the decreased expression of the CXorf56 protein following treatment of BV2 cells for 24 hrs with 25 *μ*g/ml nApoE4_1-151_. Beta-actin was employed as a loading control utilizing an anti-beta-actin antibody (1 : 50K), bottom panel. (c) Quantitative densitometry analysis of 3 independent experiments showing an overall 74% decrease in the expression of CXorf56 protein following treatment (∗ denotes *p* = 5.76 × 10^−7^). Data are representative of 3 independent experiments ± S.D. (d, e) BV2 cells were left untreated (d) or treated for 24 hours with nApoE4_1-151_ and stained with the CXorf56 antibody. Following treatment, there was a decrease in the staining intensity as well as a morphological shift to bipolar, extended pseudopods (arrowheads, e).

Next, we sought to determine a role for apoE4 in activating microglia using BV2 cells as a model system. We recently demonstrated a key role for an amino-terminal fragment of apoE4 1-151 (nApoE4_1-151_) localizing to the nucleus of microglia in the human AD brain [8]. In addition, treatment of BV2 microglia cells with nApoE4_1-151_ leads to an uptake and trafficking of nApoE4_1-151_ to the nucleus [8, 15]. We hypothesized that this fragment may be acting as a transcription factor changing the expression of proteins that may promote the inflammation associated with AD. To test this directly in the current study, we incubated BV2 microglia cells with 25 *μ*g/ml nApoE4_1-151_ or an equivalent concentration of full-length apoE4 and assessed any morphological and/or functional changes that may result in connection with CXorf56. This concentration of nApoE4_1-151_ is within the range of the normal concentration of apoE that has been documented in the human brain (3-5 *μ*g/ml) [20]. A concentration of 25 *μ*g/ml was chosen because we obtained inconsistent results with lower concentrations of nApoE4_1-151_, and overall, 10 *μ*g/ml nApoE4_1-151_ did not lead to a decrease in the level of the CXorf56 protein (see below). Treatment of BV2 microglia cells with nApoE4_1-151_ resulted in a significant downregulation of the CXorf56 homologue mRNA (blue bar, Figure 5(a)) while full-length apoE4 had no significant effect (red bar, Figure 5(a)). In addition, we observed a 73.5% decrease in the expression of the CXorf56 protein as demonstrated by Western blot analysis following incubation of BV2 cells with a 25 *μ*g/ml nApoE4 fragment, while 10 *μ*g/ml had no significant effect (Figures 5(a)–5(c)). Confocal immunofluorescence microscopy demonstrated a decrease in the staining intensity of CXorf56 following treatment with nApoE4_1-151_ (Figures 5(d) and 5(e)). Moreover, there was a clear shift in the morphology of cells that was similar to what was observed following CXorf56 knockdown with siRNA (arrowheads, Figure 5(e)); cells became elongated and bipolar, sending out long pseudopods. The actions of nApoE4_1-151_ were independent of cell toxicity and subsequent degradation of the CXorf56 protein as assessed by the release of LDH in media by BV2 microglia cells (Fig.  S3). These data suggest that nApoE4_1-151_ may promote a morphological shift of microglia cells via downregulation of the CXorf56 protein.

### 3.4. An Amino-Terminal Fragment of apoE4 Specifically Binds to the TNF*α* Promoter Region and Leads to Increased Secretion of TNF*α* and IL-1beta

**Figure 6:**
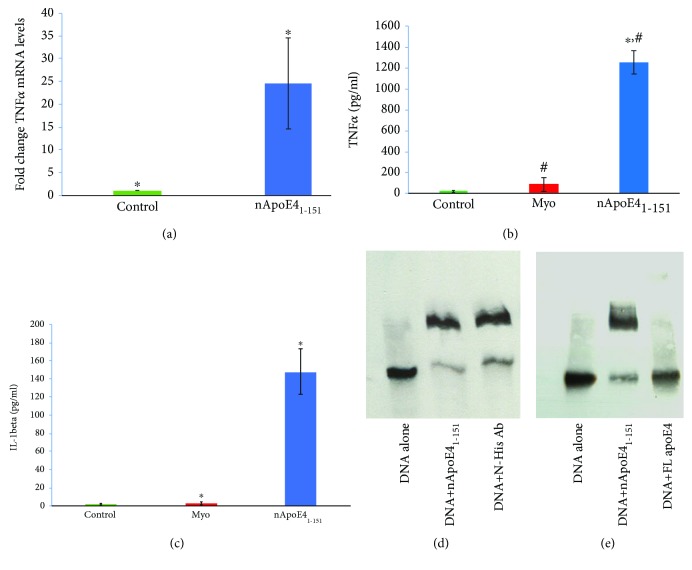
Treatment of BV2 microglial cells with an amino-terminal fragment of apoE4 leads to the upregulation of inflammatory cytokines. (a) BV2 cells were left untreated (control, green bar) or treated for 5 hours with 25 *μ*g/ml nApoE4_1-151_ (blue bar), and RNA was extracted. RT-PCR analysis indicated a 24.5-fold increase in the expression of inflammatory cytokine, TNF*α*. ∗ denotes significant difference, *p* = 0.016. (b) Secreted TNF*α* levels are significantly elevated following the treatment of BV2 cells with nApoE4_1-151_ (blue bar) as compared to untreated controls (green bar) or an unrelated protein, myoglobin, of similar size and weight to nApoE4_1-151_ (red bar, middle). Data are representative of 3 independent experiments ± S.E.M.∗ denotes significant difference, *p* = 3.8 × 10^−8^ between control and nApoE4_1-151_, while # represents significant difference, *p* = 0.00075 between myoglobin and nApoE4_1-151_. (c) Secreted IL-1beta levels are significantly elevated following the treatment of BV2 cells with nApoE4_1-151_ (blue bar) as compared to untreated controls (green bar) or an unrelated protein, myoglobin, of similar size and weight to nApoE4_1-151_ (red bar, middle). Data are representative of 3 independent experiments ± S.E.M.∗ denotes significant difference, *p* = 0.013. (d, e) DNA gel mobility shift assay was used to determine a specific interaction of nApoE4_1-151_ with the 200 bp promoter region of mouse TNF*α*. (d) Biotinylated end-labeled DNA corresponding to the mouse TNF*α* promoter region was incubated alone or in addition of 25 *μ*g/ml of nApoE4_1-151_. There was an upward shift in the DNA band indicating a binding of the fragment to DNA. The last lane was performed similarly except that the nApoE4_1-151_ fragment was preincubated first with an anti-His antibody that specifically recognizes the His-tagged nApoE4_1-151_ fragment. In this case, although shift was still evident, incubation with the anti-His antibody led to a decrease in gel retardation suggesting a specific competition between the DNA and antibody for the nApoE4_1-151_ fragment. (e) Identical experiments with the exception of an identical concentration of full-length apoE4 (25 *μ*g/ml) were tested. As denoted in the last lane, in this case, full-length apoE4 had no effect on the DNA shift suggesting that gel retardation of the TNF*α* DNA promoter region is due to specific binding to the nApoE4_1-151_ fragment.

Activated microglia express proinflammatory cytokines such as tumor necrosis factor alpha (TNF*α*) as well as IL-1beta, which are well-known signature cytokines of microglia activation. Treatment of BV2 microglia cells with nApoE4_1-151_ leads to a 24.5-fold increase in TNF*α* expression (Figure 6(a)). Paralleling the mRNA expression, we documented a significant, 68-fold increase in the level of secreted TNF*α* protein in the media following treatment of BV2 microglia cells with nApoE4_1-151_ (Figure 6(b)). As a control, we also tested whether this was related directly to the apoE4 fragment by testing myoglobin that consists of roughly the same number of amino acids and molecular weight. Myoglobin consists of 153 amino acids and has a molecular weight of 16.7 kDa. As shown in Figure 6(b), exposure of BV2 cells to myoglobin at the same concentration as to nApoE4_1-151_ did not lead to an increase in secreted TNF*α* (red bar, Figure 6(b)). Moreover, we noticed qualitatively no change in the morphology with myoglobin as compared to the nApoE4_1-151_. Similar to the results with TNF*α*, we also observed a 59-fold increase in the secretion of IL-1beta following treatment of BV2 cells with nApoE4_1-151_ (Figure 6(c)), with little effect observed following treatment with myoglobin. These results suggest a specific action of nApoE4_1-151_ on BV2 cells versus a more general microglial response.

To test whether the nApoE4_1-151_ fragment interacts specifically with the TNF*α* DNA promoter region, gel mobility shift assays were performed. Biotinylated DNA corresponding to the upstream 200 bp promoter region of TNF*α* in the mouse was incubated with nApoE4_1-151_, and a significant degree of DNA retardation was observed (Figure 6(d)). This shift was somewhat attenuated by preincubating the nApoE4_1-151_ fragment with an anti-His tag antibody that specifically recognizes the amino-terminal fragment of nApoE4_1-151_ (far right panel, Figure 6(d)). In a final set of experiments, we directly compared the ability of full-length apoE4 to bind to the TNF*α* promoter region. As shown in Figure 6(e), in contrast to the nApoE4_1-151_ fragment, full-length apoE4 did not lead to a mobility shift, suggesting that the binding of nApoE4_1-151_ is a specific event. Taken together, these results suggest an activation of microglia by nApoE4_1-151_ that occurs at least in part by changes in gene expression of CXorf56 and TNF*α*.

## 4. Discussion

A central question in AD is determining how harboring the *APOE4* allele translates molecularly into an increased risk for dementia. One possibility is the apoE4 protein may lead to enhanced inflammation that has long been documented in the AD [7, 21–25]. Emerging data suggests that apoE4 may promote pathology associated with AD through a gene-regulating mechanism. Thus, data from our lab and others have documented a nuclear localization for apoE4 that may lead to gene expression changes [15]. Recently, we demonstrated nuclear localization of an amino-terminal fragment of apoE4 nApoE4_1-151_ within microglia of the human AD brain and within BV2 microglia cells [8, 15]. BV2 cells are a well-characterized, extensively employed model system for microglia. Studies have demonstrated that BV2 cells are a valid substitute for primary microglia in many experimental settings, including complex cell-cell interaction studies [26]. For example, this study showed that in response to LPS, 90% of genes induced by the BV2 cells were also induced by primary microglia. In the current study, employing BV2 microglia cells as a model system, we tested the hypothesis that the link between harboring the *APOE4* allele and inflammation in AD may be activation of microglia by altering the expression of specific proinflammatory genes.

In the present study, we identified a novel protein, CXorf56, as a potential link between apoE4 and microglia activation. Very little is known about the *CXorf56* gene or the protein other than it has been recently linked to an inherited form of intellectual disability [16]. Our results demonstrate that the CXorf56 protein resides specifically within the ER of BV2 microglia and in liver microsomes. In addition, knockdown of CXorf56 expression led to a distinct morphological shift of BV2 microglia cells to an activated phenotype. Our data suggest that CXorf56 is required to maintain microglia cells in a round, compact morphology with little ramification. How downregulation of the CXorf56 protein leads to a morphological shift is not known, but it may occur through the ER stress pathway. Thus, several studies have linked CNS inflammation mediated in part by the activation of microglia through the ER stress pathway [27–30]. The ER stress response constitutes a cellular process that is stimulated by a variety of conditions that disturb the normal folding of proteins in the ER. Eukaryotic cells have developed an adaptive mechanism referred to as the unfolded protein response (UPR), which is aimed at clearing unfolded proteins and restoring ER homeostasis [31]. It may be that the function of CXorf56 is somehow delegated to maintaining the functioning of UPR, although future studies will be needed to assess this hypothesis.

A key outcome of the present study was identifying a novel pathway by which an amino-terminal fragment of apoE4 (nApoE4_1-151_) may lead to microglia activation. Although it is well established that harboring the *APOE4* allele enhances dementia risk, the molecular mechanisms underlying this risk has not been well established. Recent studies from our lab have supported a novel pathway for nApoE4_1-151_ whereby localization within the nucleus may lead to gene expression changes [8, 15]. In the present study, we now demonstrate that nApoE4_1-151_ leads to the downregulation of the murine CXorf56 homologue mRNA and protein resulting in a morphological shift that mirrored the downregulation of CXorf56 by siRNA constructs. Additionally, we show that treatment of BV2 microglia cells with nApoE5_1-151_ led to a 24.5-fold increase in mRNA TNF*α* expression, a potent proinflammatory cytokine. A corresponding 68-fold increase of the secreted TNF*α* protein was detected in the media following ELISA analysis. We also observed a parallel 59-fold increase in the secretion of the IL-1beta cytokine. Finally, we demonstrate a specific interaction of the nApoE4_1-151_ fragment with the mouse TNF*α* promoter region. In contrast, no specific interaction of full-length apoE4 with this promoter region was observed. In pathological conditions, microglia produce large amounts of TNF*α* that serves as an important component of neuroinflammatory response that is associated with several neurological disorders including AD [32–34]. In addition, the secretion of TNF*α* and IL-1beta serves as a classic signature for microglia activation as has been observed in AD [35]. It is noteworthy that in our studies we treated cells with a concentration of 25 *μ*g/ml of nApoE4_1-151_. In the previous study, the levels of apoE4 in the cerebral spinal fluid (CSF) were measured as being approximately 3-5 *μ*g/ml [20]. Also, we observed inconsistent effects of 10 *μ*g/ml nApoE4_1-151_ on the downregulation of the CXorf56 protein, and overall, this concentration of nApoE4_1-151_ had no statistical effect on the downregulation of the CXorf56 protein. Although the concentration of nApoE4_1-151_ used was within an order of magnitude, future studies should examine whether lower concentrations of the nApoE4_1-151_ fragment lead to significant changes in cytokine levels in order to put our current findings in a more physiological context.

We also examined whether downregulation of the CXorf56 protein led to changes in TNF*α* levels similar to what was observed for the nApoE4 fragment. Following treatment of cells with siRNA to CXorf56, we actually observed a slight decrease in the TNF*α* mRNA levels (82% of control levels). These results suggest that the CXorf56-associated changes in morphology are distinct from the upregulation of TNF*α* and that these two separate phenomena are associated with nApoE4_1-151_ fragment exposure but are not necessarily directly related.

## 5. Conclusions

Collectively, these data suggest that apoE4 may promote the activation of microglia by a gene-regulatory mechanism involving a newly identified protein, CXorf56 as well as TNF*α*. These results suggest a novel role for apoE4 and provide a possible link to the inheritance of this allele to inflammation associated with AD. Future studies should be directed towards replicating these findings in primary microglia cultures or in induced pluripotent stem cells.

## Figures and Tables

**Table 1:** 

Gene	Oligo sequences (5′-3′)	*E*%
SFRS11	AAATACCACCCAACAGTTT	101
	AAGCCCTATACAGATGGAT	
EHBP	GAGTCTCCAATATCATCAGTAAGC	96
	ACACATGCCACGATCAATG	
CXorf56	GTGTTGCCAGGAAATAGTTTTCTTC	109
	CCCAGGATCGCTAAC	

## Data Availability

The data used to support the findings of this study are included within the article.

## Supplementary Materials

Supplementary Materials Fig. S1: confocal labeling of two different markers in BV2 microglial cells. The green image in (A) is showing the use of the cytochrome C antibody, the red label is showing the use of the CXorf56 antibody (B), and (C) is showing the overlap image. Fig. S2: representative Western blot analysis using the CXorf56 antibody in increasing amounts of liver microsomes. The data show the presence of a single 34 kDA band corresponding to the molecular weight of native CXorf56. Fig. S3: quantitative analysis of BV2 microglia toxicity by measuring the release of LDH in the media. The data show untreated, control cells (blue bar) and the following treatment with the apoE4 1-151 amino-terminal fragment (orange bar). There was no significant difference in cytotoxicity following treatment.
