# Supplementary material for: A Fragment of Apolipoprotein E4 Leads to the Downregulation of a CXorf56 Homologue, a Novel ER-Associated Protein, and Activation of BV2 Microglial Cells
Source: Oxid Med Cell Longev. 2019 May 6;2019:5123565. doi: 10.1155/2019/5123565 (PMC6526552; doi:10.1155/2019/5123565)

Supplementary Figures:

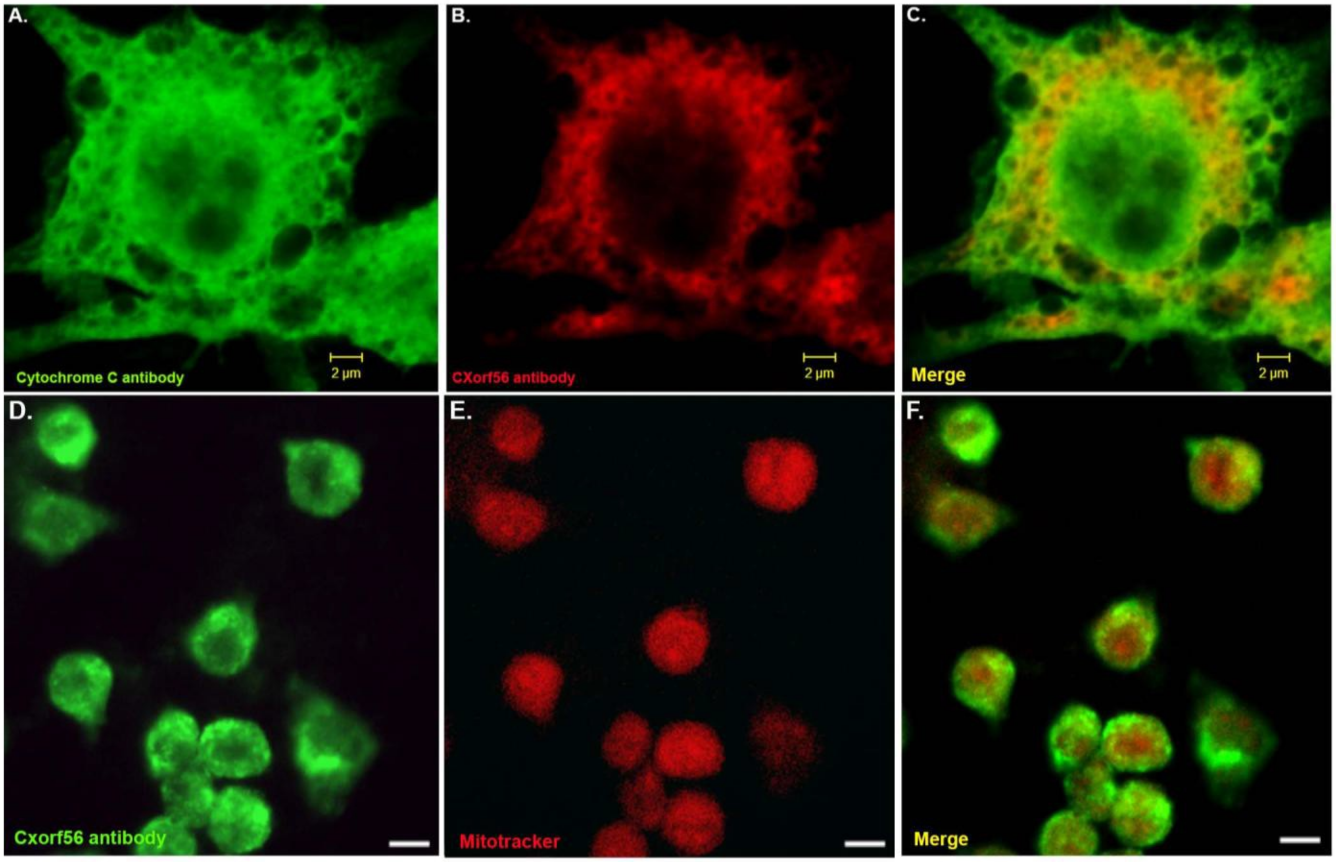

Fig. S1. The CXorf56 protein does not co-localize with the mitochondrial specific marker, cytochrome C or Mitotracker dye in BV2 cells. (A-C): BV2 cells, representing a microglia cell line, were plated on glass chamber slides in normal growth media for 24 hours. Cells were fixed and double-label confocal microscopy was carried out using a monoclonal antibody to cytochrome C (A, green), or a rabbit polyclonal antibody to CXorf56 (B, red), with the overlap image shown in (C). Little co-localization of these two markers was apparent in the overlap imaged as evidenced by the lack of strong yellow labeling. Data are representative of 3 independent experiments. (D-F): BV2 cells were plated on glass chamber slides in normal growth media for 24 hours and then incubated with the live stain, Mitotracker Deep Red FM (Life Technologies,

Carlsbad, CA) (E, red) according the manufacturer's instructions. Following labeling cells were fixed and immunocytochemistry was carried out using the rabbit polyclonal antibody to CXorf56 (D, green). The overlap image is shown in Panel F and indicates very little co-localization between the two markers. Scale bars represent 10 μM.

Fig. S2. Expression of the CXorf56 protein within the ER of liver microsomes. Various concentrations of mouse liver microsomes derived from the ER of hepatic cells were separated onto 15% SDS-PAGE gels, transferred to nitrocellulose, and then probed with the CXorf56 antibody (1:500). The results indicated strong immunoreactivity of the CXorf56 antibody to a single band corresponding to the correct, predicted molecular weight for the CXorf56 protein at 34 kDa.

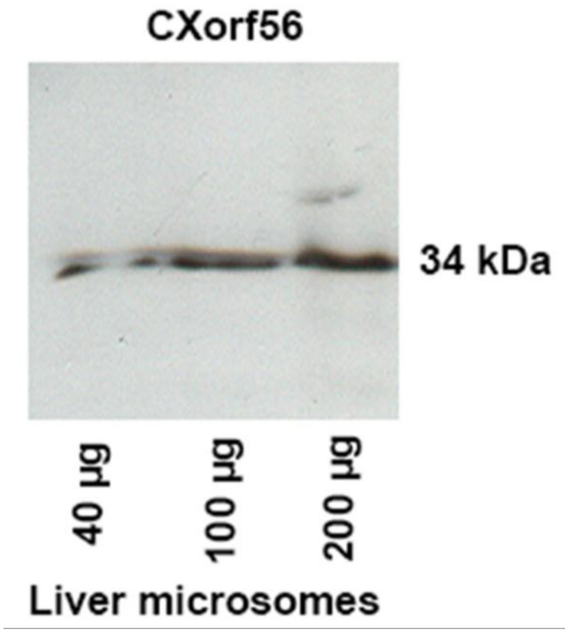

Fig. S3. Assessment of cytotoxicity by an amino-terminal fragment of apoE4. BV2 cells were cultured in sterile 6-well plates until confluent and then left untreated (blue bar) or treated with 25 µg/ml nApoE4$_{1-151}$ (orange bar) for 24 hours. Following treatment, the supernatant from each condition was collected and analyzed for LDH activity as described in the Materials and Methods. No significant difference in cytotoxicity was observed following treatment of cells with nApoE4$_{1-151}$.

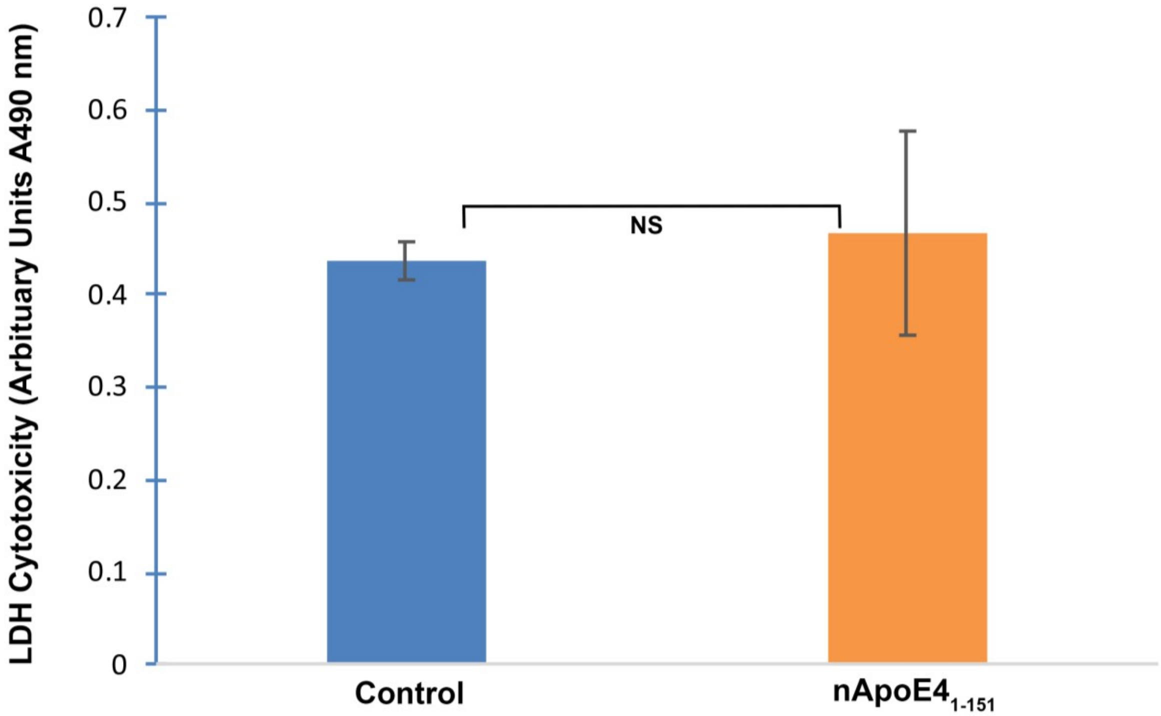

Supplement: Supplementary Materials — Fig. S1: confocal labeling of two different markers in BV2 microglial cells. The green image in (A) is showing the use of the cytochrome C antibody, the red label is showing the use of the CXorf56 antibody (B), and (C) is showing the overlap image. Fig. S2: representative Western blot analysis using the CXorf56 antibody in increasing amounts of liver microsomes. The data show the presence of a single 34 kDA band corresponding to the molecular weight of native CXorf56. Fig. S3: quantitative analysis of BV2 microglia toxicity by measuring the release of LDH in the media. The data show untreated, control cells (blue bar) and the following treatment with the apoE4 1-151 amino-terminal fragment (orange bar). There was no significant difference in cytotoxicity following treatment. [file 5123565.f1.pdf]
